# Parietal subdural empyema as complication of acute odontogenic sinusitis: a case report

**DOI:** 10.1186/1752-1947-8-282

**Published:** 2014-08-21

**Authors:** Francesco Martines, Pietro Salvago, Sergio Ferrara, Marianna Mucia, Angelo Gambino, Federico Sireci

**Affiliations:** 1 Audiology Section, Department of Biopathology and Medical and Forenses Biotechnologies (DiBIMEF), University of Palermo, Palermo, Italy; 2 Otorhinolaryngology Section, Department of Experimental Biomedicine and Clinical Neurosciences (BioNeC), University of Palermo, Palermo, Italy; 3 Radiology Section, Department of Biopathology and Medical and Forenses Biotechnologies (DiBIMEF), University of Palermo, Palermo, Italy

**Keywords:** Intracranial infections, Odontogenic sinusitis, Paranasal sinusitis, Subdural empyema

## Abstract

**Introduction:**

To date intracranial complication caused by tooth extractions are extremely rare. In particular parietal subdural empyema of odontogenic origin has not been described. A literature review is presented here to emphasize the extreme rarity of this clinical entity.

**Case presentation:**

An 18-year-old Caucasian man with a history of dental extraction developed dysarthria, lethargy, purulent rhinorrhea, and fever. A computed tomography scan demonstrated extensive sinusitis involving maxillary sinus, anterior ethmoid and frontal sinus on the left side and a subdural fluid collection in the temporal-parietal site on the same side. He underwent vancomycin, metronidazole and meropenem therapy, and subsequently left maxillary antrostomy, and frontal and maxillary sinuses toilette by an open approach. The last clinical control done after 3 months showed a regression of all symptoms.

**Conclusions:**

The occurrence of subdural empyema is an uncommon but possible sequela of a complicated tooth extraction. A multidisciplinary approach involving otolaryngologist, neurosurgeons, clinical microbiologist, and neuroradiologist is essential. Antibiotic therapy with surgical approach is the gold standard treatment.

## Introduction

Suppurative intracranial infections (meningitis, intracranial abscess, subdural empyema and epidural abscess) are uncommon sequelae of paranasal sinusitis. In fact, the incidence of morbidity and mortality has been reported to range from 5 to 40% [1-4]; this is because the diagnosis is often unsuspected [5].

The literature on intracranial complications of sinusitis consists mainly of case reports with the exception of a few large series of hospitalized patients that present a rate of intracranial complications that varies from 3.7% to 47.6% [2,6,7].

However, paranasal sinuses disease is the presumed underlying cause of approximately 10% of intracranial suppuration [3,7].

The frontal lobe is the most common location, and it is usually caused by chronic frontal sinusitis associated to nasal polyposis. Parietal lobe abscesses are usually associated with sphenoid rhinosinusitis, whereas there is rarely a correspondence to a temporal lobe abscess.

To date there is no evidence of an intracranial complication caused by tooth extraction. In fact, based on clinical presentation and microbiology, odontogenic paranasal sinus infections usually can be differentiated from those attributed to upper respiratory tract infections [8]; odontogenic paranasal sinus infections cause swelling in one or more of the deep fascial spaces of head and neck.

We report a rare and insidious case of parietal subdural empyema evolving over 2 weeks, secondary to dental extraction.

## Case presentation

**Figure 1:**
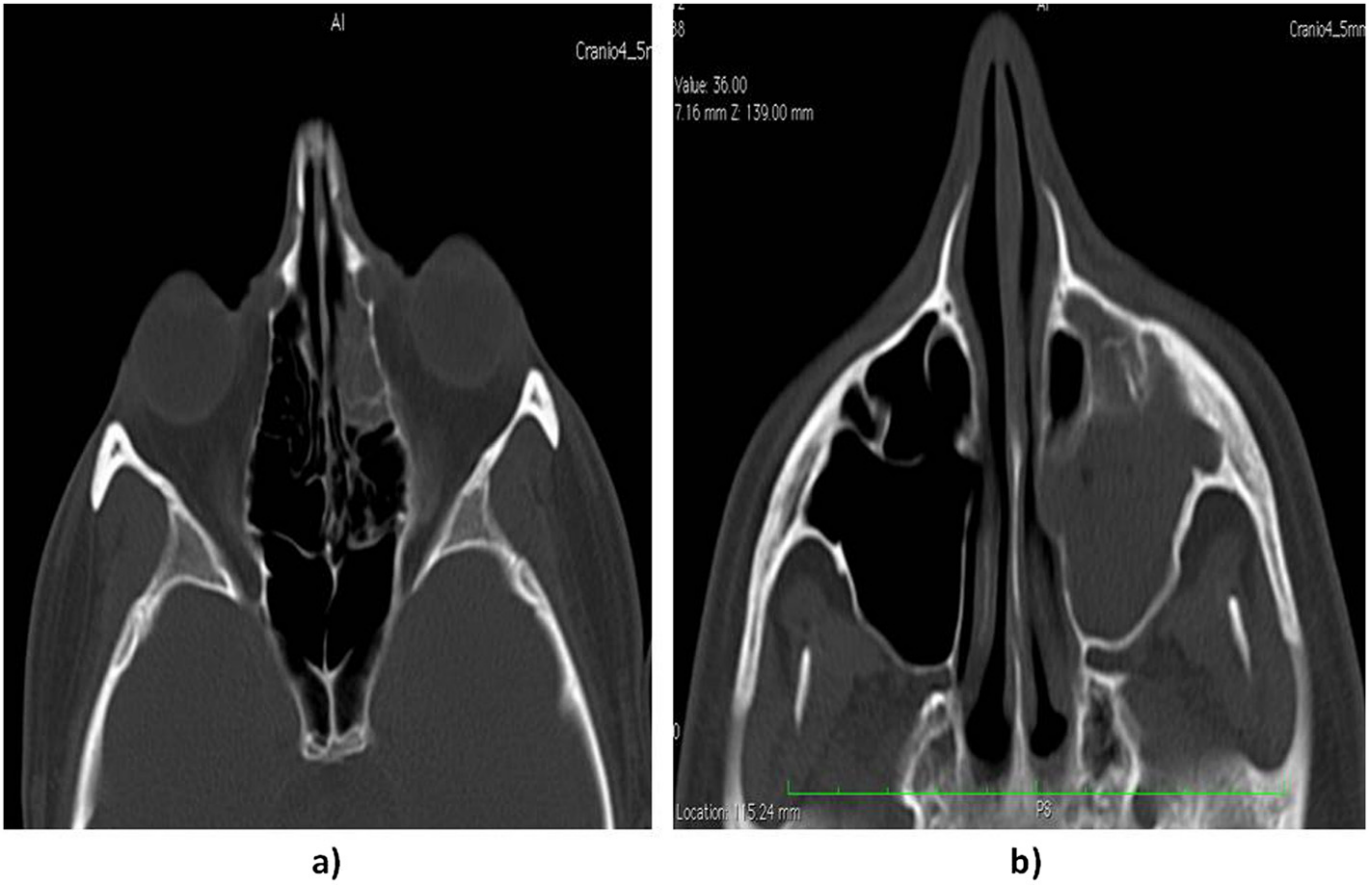
Axial computed tomography scans showing the left anterior ethmoid (a) and left maxillary sinus (b) involvement by the infective process.

**Figure 2:**
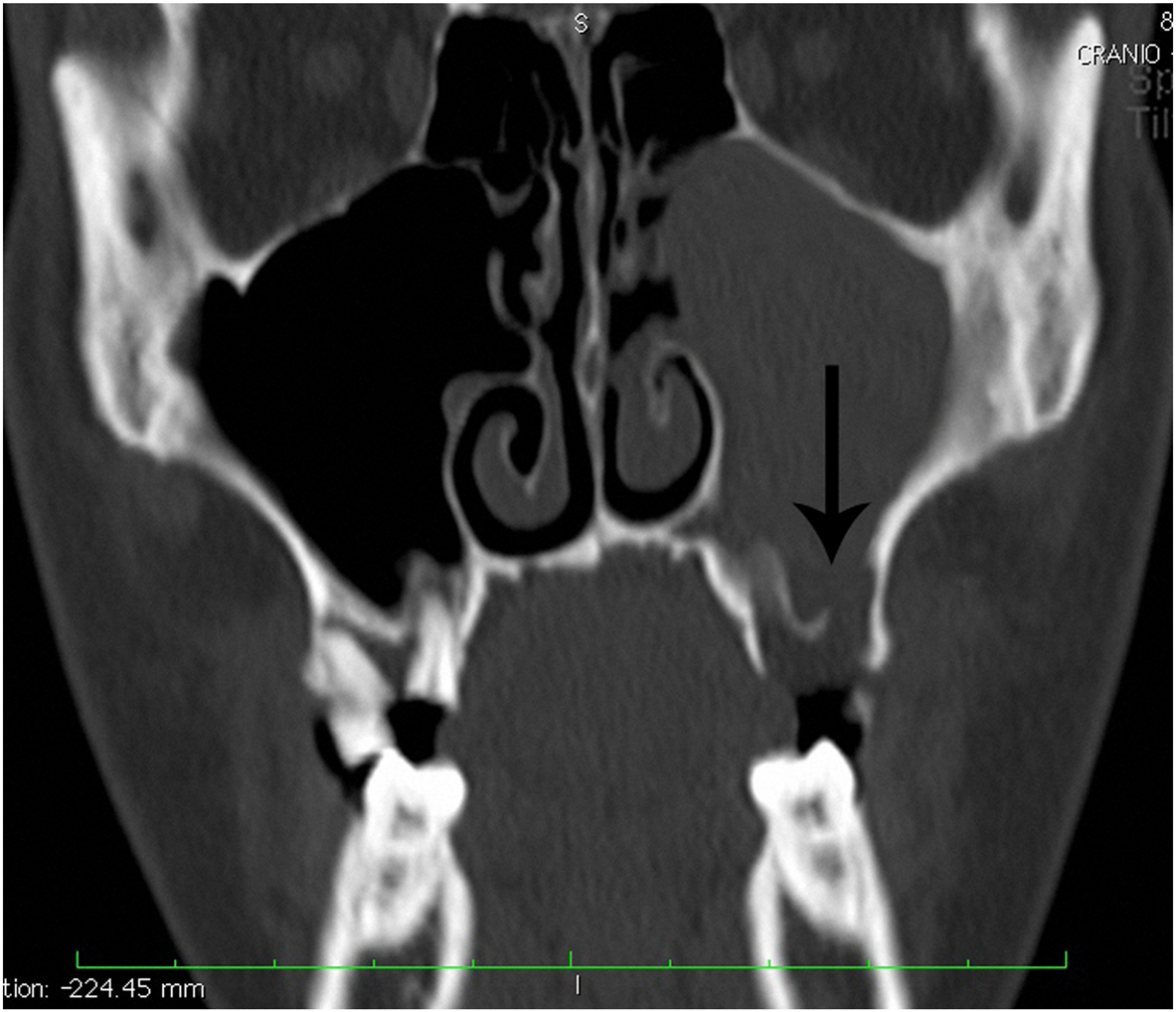
Coronal computed tomography scan showing the interruption of the sinus floor (black arrow) corresponding to alveolar process secondary to tooth extraction.

**Figure 3:**
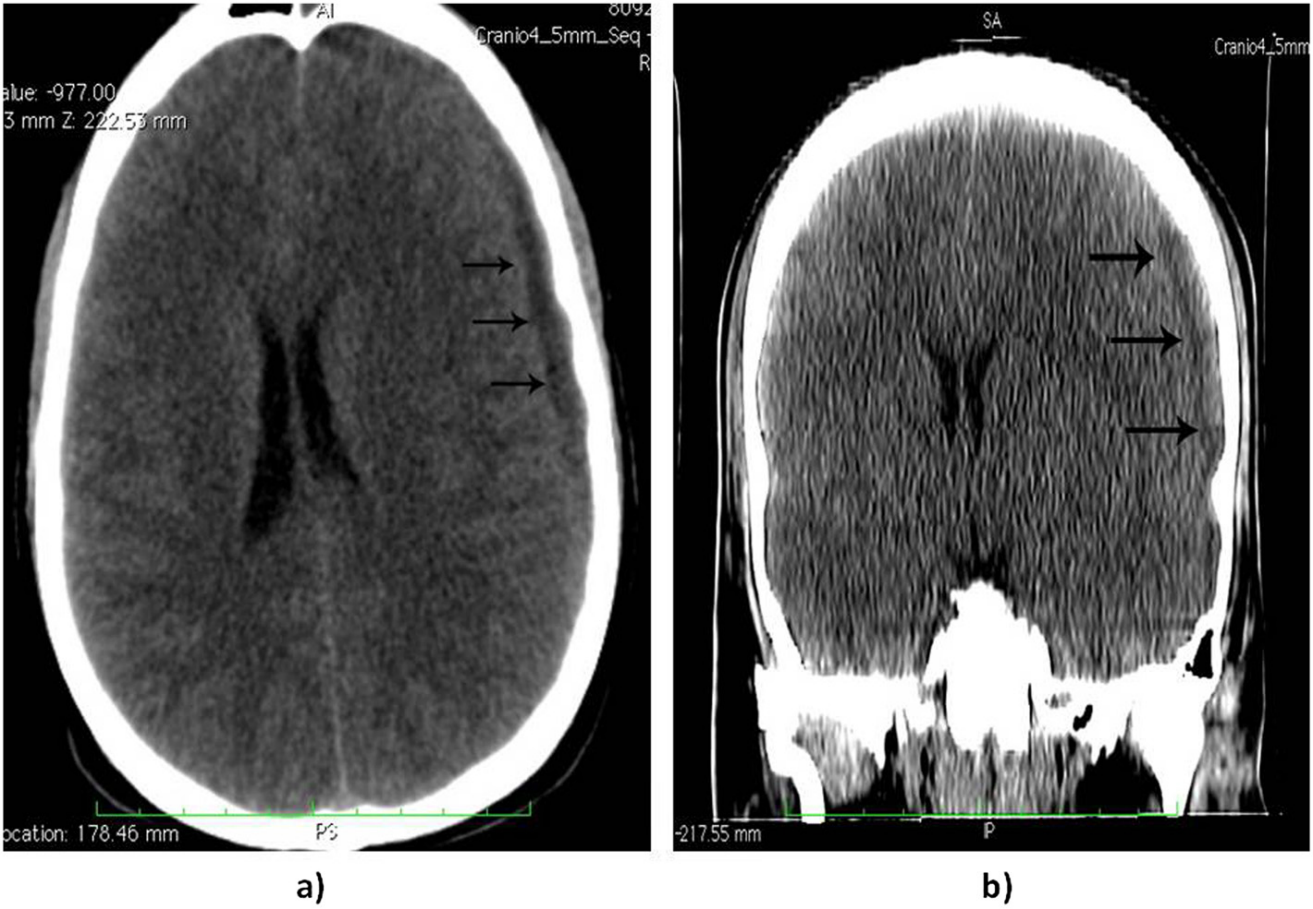
**Computed tomography scan in axial (a) and coronal (b) cut: displaying the subdural collection (black arrows) of exudates on the left side in the temporal-parietal region.** The air content of the empyema is noticeable.

An 18-year-old Caucasian man was admitted to our Institution because of dysarthria, lethargy, purulent rhinorrhea, and fever for 2 days. His 6th dental element of the left side had been extracted 10 days earlier. His past medical history was unremarkable; he had a positive remote history for post-traumatic splenectomy. The first clinical examination revealed an increased body temperature of 37.6°C, heart rate of 82 beats per minute, blood pressure 150/64mmHg, and respiratory rate of 16 breaths/minute. He was orientated, his cranial nerves were intact, and his pupils were reactive, round and equal. His white cell count was 30,620 per μL with 80.2% of neutrophils and 6.6% lymphocytes. His hemoglobin was 11.1g/dL and C-reactive protein was 80mg/L. The levels of his chemistries were normal. His neurological examination confirmed the lethargic status and the dysarthria, whereas the conscious alertness was maintained. An anterior rhinoscopy displayed the presence of pus filling his whole nasal cavity, which was originating from the middle meatus. An examination of his oral cavity showed a fistula in the site of dental extraction communicating with the maxillary sinus. The findings of our physical examination of his head and neck were not significant; in particular there were no lymph nodes clinically evident.A computed tomography (CT) scan without contrast enhancement in emergency was performed and showed extensive sinusitis involving maxillary sinus, anterior ethmoid and frontal sinus on the left side (Figure 1). An interruption of sinus floor corresponding to alveolar process was evident (Figure 2). The orbital content was intact. A subdural fluid collection was present in the temporal-parietal site on the left side; a mass effect was evident with displacement of the middle line structures toward the opposite side (Figure 3).

He was admitted to our intensive care unit and underwent vancomycin and metronidazole therapy as standard protocol for sinusitis associated with intracranial complication. His condition deteriorated after 6 hours with right-side hemiparesis; after a further CT examination confirmed enlargement of the fluid collection, an emergent craniotomy was done to evacuate the subdural empyema. A cultural examination was attempted with the fluid evacuation and showed the massive presence of *Bacteroides* and a few colonies of alpha-hemolytic *Streptococci*, sensitive to meropenem that was added to therapy.

Two days after craniotomy, left maxillary antrostomy and frontal and maxillary sinuses toilette were performed by an open approach.

His fever decreased progressively in the next 3 days until his body temperature normalized. His cognitive conditions improved slowly and he was extubated on hospital day 10. Parenteral therapy and nutrition was continued for a further 12 days. The hemiparesis regressed progressively in 5 days. He was discharged 32 days after admission with normal alertness and gait, and a residual dysarthria; an antiepileptic therapy was prescribed with levetiracetam 1000mg per day. The last clinical control done after 3 months showed a regression of the dysarthria; a rhinoscopy revealed a good healing of the antrostomy, with normal drainage of his maxillary sinus, and the oral fistula appeared closed.

## Discussion

Various etiologies are described in the literature as *primum movens* of subdural empyema including paranasal sinusitis (Table 1) [1-7,9-22].

**Table 1:** Systematic literature review of intracranial complications of paranasal sinusitis

**Author**	**Year**	**Study design**	**Number of intracranial complications of paranasal sinusitis**	
**Reference number**				
		**Total cases reviewed**	**N**	**Involved sinuses**
Maniglia *et al*. [3]	1989	Review of intracranial infection	13	3 Pansinusitis
		19		2 Frontal
				1 Frontal/maxillary
				4 Ethmoid/frontal
				2 Ethmoid
				1 Ethmoid/sphenoid
Clayman *et al*. [2]	1991	Review of paranasal sinusitis	24	3 Pansinusitis
		649		2 Hemipansinusitis
				6 Frontal
				1 Frontal/maxillary
				1 Maxillary
				4 Bilateral frontal
				2 Ethmoid/frontal
				4 Ethmoid
				1 Ethmoid/sphenoid
Dolan and Chowdhury [14]	1995	Case reports	5	1 Pansinusitis
		5		2 Frontal
				1 Ethmoid/sphenoid
				1 Ethmoid/maxillary
Nathoo *et al*. [15]	1997	Review of intracranial infection	1	Pansinusitis
		3865		
Giannoni *et al*. [7]	1997	Review of intracranial infection	12	7 Pansinusitis
		203		1 Maxillary
				1 Ethmoid/maxillary
				1 Ethmoid/frontal
				1 Frontal/sphenoid
				1 Sphenoid
Akimura *et al*. [16]	1998	Case report	1	Pansinusitis
Gallagher *et al*. [4]	1998	Review of intracranial infection	15	5 Pansinusitis
		176		5 Hemipansinusitis
				1 Ethmoid/sphenoid
				3 Frontal
				1 Ethmoid
Sahjpaul and Lee [17]	1999	Case report	1	Pansinusitis
Chandy *et al*. [11]	2001	Case report	1	Ethmoid/maxillary/frontal
Younis *et al*. [6]	2002	Review of sinusitis complication	39	5 Ethmoid/sphenoid
		82		25 Pansinusitis
				9 Hemipansinusitis
Moonis *et al*. [5]	2002	Case report	1	Sphenoid
Jones *et al*. [1]	2002	Review of intracranial infection secondary to sinusitis	47	18 Ethmoid/frontal
				24 Frontal
		47		2 Sphenoid
				3 Ethmoid
Lu *et al*. [18]	2002	Review of intracranial infection	9	–
		123		
Unlu *et al*. [19]	2002	Case report	1	Sphenoid
Roche *et al*. [20]	2003	Review of intracranial infection	35	–
		163		
Fountas *et al*. [20]	2004	Case report	2	1 Frontal
				1 Ethmoid/frontal
Ali A *et al.*[30]	2005	Review of acute rhino-sinusitis complication	3	2 Ethmoid/frontal
				1 Frontal
		13		
Gois *et al*. [23]	2005	Review of intracranial complication secondary to sinusitis	21	–
		21		
Betz CS *et al.*[31]	2008	Review of acute frontal sinusitis complication	3	3 Frontal
		12		

"–" indicate absence of information in the references.

For the first time, we present an unusual case described in literature of a parietal subdural empyema secondary to acute odontogenic sinusitis, resulting from a tooth extraction (Table 2) [2,3,7,11,18,20,23]. According to Clayman *et al*., odontogenic sinusitis accounts for approximately 10% of cases of all maxillary sinusitis [2]. The microbiology of rhinosinusitis and odontogenic maxillary sinusitis are thought to be different; in fact it is universally accepted that *Streptococcus pneumoniae*, *Haemophilus influenzae* and *Moraxella catarrhalis* are commonly associated to upper respiratory tract sinusitis, whereas the typical odontogenic infection is a mixed aerobic/anaerobic infection, with a prevalence of anaerobic species (*Streptococci*, *Bacteroides*, *Proteus*, and coliform bacilli) [24]. Literature data confirm that more than 70% of rhinosinusitis intracranial complications are caused by *Streptococci* species, 15 to 20% by *Staphylococcus aureus* and 7% by *Haemophilus* species [20].

**Table 2:** Intracranial complication of odontogenic sinusitis

**Author Reference number**	**Cases/total**	**Involved sinuses**	**Odontogenic cause**		
			**Diagnosis**	**Site of complication**	**Notes**
Maniglia *et al*. [3]	1/13	Pansinusitis	Confirmed	Multiple subdural empyema	*Bacteroides*; Dead
Clayman *et al*. [2]	2/24	Frontal/maxillary	Probable	Meningitis	–
		Maxillary	Probable	Multiple septic embolic infarcts	–
Giannoni *et al*. [7]	1/12	Maxillary	Probable	–	–
Chandy *et al*. [11]	1	Ethmoid/maxillary/frontal	Confirmed	Pott’s puffy tumor and epidural abscess	Dental extraction 2 months before
Lu *et al*. [18]	1/9	–	Probable	Brain abscess	*Bacteroides*
Roche *et al*. [20]	2/35	–	Confirmed	Brain abscess	–
Martines *et al*. [22]	1	Ethmoid/maxillary/frontal	Confirmed	Parietal subdural empyema	*Bacteroides*

"–" indicate absence of information in the references.

As our cultural examination revealed, the presence of *Bacteroides* in the subdural empyema was indicative of a relationship between such complication and the recent tooth extraction.

The roots of the maxillary premolar and molar teeth are situated below the sinus floor; in particular the second molars roots are the closest to the floor (mean distance of 1.97mm) [25]. These short distances explain how oroantral fistula may be responsible for the development of maxillary sinusitis that is generally characterized by a chronic course.Our case is remarkable for numerous reasons. He did not report a history of chronic sinusitis at the initial examination and, furthermore, there were no clinical or radiographic signs of maxillary sinusitis on the contralateral sinus. The time interval between acute sinusitis (purulent rhinorrhea and nasal obstruction) and symptoms of intracranial diseases, such as headache, altered mental status and lethargy, was of 2 days. It led us to believe that the sinusitis was of an acute type and was initiated by iatrogenic opening of an oroantral fistula (Figure 2).

The predisposition of young men to develop empyema has been explained by the high vascularity of the diploic system in this age group [2]. Moreover, in our patient the absence of the spleen was probably a cofactor of the rapid development of the intracranial complication. Finally, the presence of the air content into subdural space is suspicious of the odontogenic anaerobic microflora that was founded at cultural examination after empyema evacuation and that represents an extremely rare report.

Radiological evaluation should be done in all patients in whom subdural empyema is suspected. Although magnetic resonance imaging (MRI) is more sensitive in showing parenchymal abnormalities such as abscess [13], cranial CT is often the first neuroimaging done in emergency and gold standard for visualization of the paranasal sinuses and associated bony abnormalities. In early cases of subdural empyema, CT might not show a fluid collection [26-28], so consideration should be given to repeated CT imaging or MRI as the clinical scenario dictates. As shown by our case, CT evidenced the empyema as a thin, hypodense subdural lesion, with linear enhancement of the medial surface (Figure 3a and b). The grey-matter/white-matter interface is displaced inwardly. Mass effect is generally caused by edema and ischemia rather than mass effect from the abscess. The edema can cause effacement of the basilar cisterns and flattening of the cortical sulci [27]. The sinuses might appear opacified, with air-fluid levels and bony erosion evident in some cases [27], but a clinical report of air levels into subdural space is extremely rare [13].

According to our neurosurgeon’s diagnosis, he did not undergo lumbar puncture because it is hazardous and contraindicated in patients with subdural empyema, particularly if mass effect is present on CT [28]. In fact, the results are not specific and a neurological deterioration and transtentorial herniation after lumbar puncture are well described, and have led to death [1,26,28].

Various therapeutic measures include intravenous antibiotics, craniotomy drainage of intracranial abscess, and endoscopic and/or external drainage of affected sinuses. Once the diagnosis is made, the patient must undergo a combination of high-dose antimicrobial therapy that should be directed against the most common organisms and should include broad-spectrum activity against aerobic and anaerobic cocci and bacilli. Recommended empiric therapy is a third-generation cephalosporin plus metronidazole, which offers broad coverage and good cerebrospinal fluid and abscess penetration [20]. In our case, according to our neurosurgeon, he was given vancomycin and metronidazole and the antibiotic regimen was modified after the culture reports with the adding of meropenem, which is the gold standard therapy in cases of *Bacteroides*[24]. However, it is universally accepted that aggressive antibiotic therapy is not an alternative to surgical drainage that is recommended without delay. The goals of surgical intervention are both decompression of the brain and complete evacuation of purulence through craniotomy [29], and a definitive management of the infected sinuses should be done. The choice of surgical approach depends on the involved sinus and can include maxillary irrigation, external frontoethmoidectomy, sphenoid sinusotomy, antral washout, and frontal trephine. With the advent of endoscopy in treatment of sinusitis, the external approach has been less utilized [23]; according to some authors [3,24,25,30,31], we adopted the external approach for a complete curettage of the maxillary sinus.

## Conclusions

The incidence of intracranial complications of sinusitis has decreased, but subdural empyemas remain an uncommon, but important, clinical condition with a variable incidence of morbidity and mortality. The occurrence of such disease is possible even in the case of a complicated tooth extraction that usually is responsive to standard medical and surgical treatments. The astute clinician should consider such complications in any patient presenting with fever, headache, and neurological deficits. A multidisciplinary approach involving otolaryngologist, neurosurgeons, clinical microbiologist, and neuroradiologist is essential. Effective management requires prompt diagnosis, surgical intervention in most cases, and appropriate antibiotics therapy.

## Consent

Written informed consent was obtained from the patient for publication of this case report and accompanying images. A copy of the written consent is available for review by the Editor-in-Chief of this journal.

## Abbreviations

CT: Computed tomography; MRI: Magnetic resonance imaging.

## Competing interests

The authors declare that they have no competing interests in the preparation of this article.

## Authors’ contributions

FM was the major contributor to writing the manuscript; PS, collection of data, manuscript preparation; SF, collection of data; MM, manuscript preparation, analysis; AG, analysis and collection of data; FS, manuscript preparation and analysis. All authors read and approved the final manuscript.
